# Sappanone a prevents diabetic kidney disease by inhibiting kidney inflammation and fibrosis *via* the NF-κB signaling pathway

**DOI:** 10.3389/fphar.2022.953004

**Published:** 2022-08-16

**Authors:** Zhe Wang, Zhida Chen, Xinyi Wang, Yepeng Hu, Jing Kong, Jiabin Lai, Tiekun Li, Bibi Hu, Yikai Zhang, Xianan Zheng, Xiaoxian Liu, Shengyao Wang, Shu Ye, Qiao Zhou, Chao Zheng

**Affiliations:** ^1^ Department of Endocrinology, The Second Affiliated Hospital, School of Medicine, Zhejiang University, Hangzhou, China; ^2^ Department of Nephrology, The Second Affiliated Hospital, School of Medicine, Zhejiang University, Hangzhou, China; ^3^ Department of Endocrinology, The Second Affiliated Hospital and Yuying Children’s Hospital of Wenzhou Medical University, Wenzhou, China; ^4^ Department of Pathology, The Second Affiliated Hospital, School of Medicine, Zhejiang University, Hangzhou, China; ^5^ Nanjing Kingmed Center for Clinical Laboratory Co., Ltd., Nanjing, China

**Keywords:** NF-κB, nuclear factor kappa B, SA, sappanone A, diabetic kidney disease, glomerular mesangial cells, inflammation

## Abstract

**Background:** Low grade of sterile inflammation plays detrimental roles in the progression of diabetic kidney disease (DKD). Sappanone A (SA), a kind of homoisoflavanone isolated from the heartwood of *Caesalpinia sappan*, exerts anti-inflammatory effects in acute kidney injury. However, whether SA has beneficial effects on diabetic kidney disease remains further exploration.

**Methods and Results:** In the present study, uninephrectomized male mice were treated with Streptozotocin (STZ, 50 mg/kg) for five consecutive days to induce diabetes. Next, the diabetic mice were administered orally with SA (10, 20, or 30 mg/kg) or vehicle once per day. Our results showed that STZ treatment significantly enhanced damage in the kidney, as indicated by an increased ratio of kidney weight/body weight, elevated serum creatinine and blood urea nitrogen (BUN), as well as increased 24-h urinary protein excretion, whereas SA-treated mice exhibited a markedly amelioration in these kidney damages. Furthermore, SA attenuated the pathological changes, alleviated fibrotic molecules transforming growth factor-β1 (TGF-β1) and Collagen-IV (Col-IV) production, decreased inflammatory cytokines interleukin-1β (IL-1β) and tumor necrosis factor-α (TNF-α) expression in STZ-treated mice. Similarly, in glomerular mesangial cells, SA pretreatment decreased high glucose (HG)-induced proliferation, inflammatory cytokines excretion, and fibrotic molecules expression. Mechanistically, SA decreased the expression of nuclear factor kappa B (NF-κB) and restored the expression of total NF-κB inhibitor alpha (IκBα) both *in vivo* and *in vitro*.

**Conclusion:** Our data suggest that SA may prevent diabetes-induced kidney inflammation and fibrosis by inhibiting the NF-κB pathway. Hence, SA can be potential and specific therapeutic value in DKD.

## Introduction

Diabetic kidney disease (DKD), a kind of diabetic microvascular complication, represents the most leading cause of end-stage kidney disease worldwide (Lu et al., 2020). DKD is characterized by glomerular hypertrophy, glomerular basement membrane (GBM) thickening, tubulointerstitial fibrosis, and extracellular matrix (ECM) expansion (Kanwar et al., 2011; Lim, 2014). As the major components of the glomerulus, glomerular mesangial cells play a critical role in regulating glomerular structure and function (Schlondorff and Banas, 2009). Owing to the consistent stimulation of high glucose (HG), the glomerular mesangial cells exhibit inappropriate proliferation, excessive inflammatory cytokines and fibrotic molecules excretion, which exacerbate ECM accumulation and the glomerular sclerosis (Schena and Gesualdo, 2005). Although extensive researches have been done in clarifying the inflammatory and fibrotic processes, the existing classic anti-inflammatory therapy could not efficiently prevent or reverse the development of DKD (Alicic et al., 2018; Tuleta and Frangogiannis, 2021). Hence, there is an urgent need to develop novel and effective therapeutic strategies for DKD.

Accumulating evidence indicates that consistent low grade of sterile inflammation is pivotal in the pathophysiology of DKD, including proinflammatory cytokines excretion, the chemokines and adhesive molecules recruitment, as well as nuclear factor κB (NF-κB) activation (Alicic et al., 2017; Rayego-Mateos et al., 2020; Liu et al., 2021). Among which, NF-κB activation is defined as an “inflammatory signature” which triggers kidney inflammation during the progression of DKD (Navarro-Gonzalez and Mora-Fernandez, 2008; Sanz et al., 2010; Navarro-Gonzalez et al., 2011). Under normal conditions, NF-κB remains inactive in the cytoplasm by interacting with the IκB proteins (inhibitors of NF-κB). Activation of NF-κB is initiated by degradation of IκB proteins in response to extracellular signals. After IκB proteins degradation, NF-κB enters the nucleus to induce the transcription of inflammatory genes (Navarro-Gonzalez et al., 2011). Ultimately, NF-κB activation regulates the expression of adhesion molecules, pro-inflammatory cytokines and chemokines that are associated with chronic kidney inflammation in DKD (Kim and Park, 2016). Besides, it is noteworthy that activation of NF-κB pathway is also involved in the development of kidney fibrosis. For instance, suppression of NF-κB pathway significantly attenuated kidney fibrosis in DKD (Liang et al., 2018; Sun et al., 2021).

Sappanone A (SA), a kind of homoisoflavanone isolated from the heartwood of *Caesalpinia sappan*, has been reported to exhibit anti-inflammatory effects (Lee et al., 2015). The classic inflammatory processes are associated with the action and recruitment of inflammatory cells like macrophages and T cells, and the corresponding cytokines excretion. In RAW264.7 cells, a murine macrophage cell line, SA treatment inhibited LPS-induced IL-6 expression (Chu et al., 2013). Recently, SA has been shown to play a critical role in various disease such as myocardial ischemia-reperfusion injury, cerebral ischemia-reperfusion injury and acute kidney injury (Kang et al., 2016; Shi et al., 2021; Wang et al., 2021). However, there is still no direct evidence to demonstrate whether SA has beneficial effects on DKD, and the potential mechanism remains unknown.

In the present study, we first detected the reno-protective effects of SA in Streptozotocin (STZ)-treated mice. Next, we performed *in vitro* experiments to further explore the underlying mechanisms of SA in HG-treated glomerular mesangial cells. Our research indicates that SA may prevent diabetes-induced kidney inflammation and fibrosis by inhibiting the NF-κB pathway.

## Materials and methods

### Animal models and treatment protocols

All experiments were approved by the Animal Care Committee of Zhejiang University, and the animal work was carried out at Zhejiang University. Six-eight weeks old C57BL/6J mice (20–23 g bodyweight) were purchased from Silaike Company (Shanghai, China). All mice were allowed free access to food and water, under controlled temperature (22–25°C), humidity (60%), and light (alternating 12-hour light/dark cycle) conditions. Because mice with a C57BL/6 background did not develop lesions of DKD readily after the induction of diabetes by STZ, uninephrectomy was performed to hasten the development of DKD (Zhou et al., 2019). After a week of adaptive feeding, mice were fully anesthetized with their kidney exposed *via* a back incision, the left kidney was removed after ligaturing the kidney pedicle with a 4/0 surgical suture (Ahmad et al., 2019) and randomly divided into five groups (*n* = 5 per group): Control group, STZ group, STZ + SA 10 mg/kg group, STZ + SA 20 mg/kg group, and STZ + SA 30 mg/kg group. After a one-week recovery period from unilateral nephrectomy (Unx), mice rendered diabetic were induced by intraperitoneal (I.P) injection with STZ [50 mg/kg body weight dissolved in 100 mM citrate buffer (pH 4.5)] for five consecutive days. Meanwhile, mice in the Control group received the same volume of citrate buffer. At day 7, mice with a fasting-blood glucose >12 mmol/L were considered diabetes and were used for the further study (Zheng et al., 2019). Next, the diabetic mice in STZ + SA 10 mg/kg group, STZ + SA 20 mg/kg group, and STZ + SA 30 mg/kg group were orally treated with SA dissolved in 0.5% carboxymethyl cellulose at a dose of 10, 20, or 30 mg/kg SA every day, respectively. Meanwhile, mice in the Control group and STZ group were gavaged with the same volume of 0.5% carboxymethyl cellulose. The fasting blood glucose and body weight were measured every week for 12 weeks.

At the end of the experiment, mice were placed in individual metabolic cages for 24 h urine collection. The animals were then sacrificed and blood was collected from the right ventricle of the heart using a heparin-free syringe with a needle at the time of death. Blood urea nitrogen, serum creatinine, and daily urinary albumin excretion were measured using an automatic biochemical analyzer (Hitachi, Tokyo, and Japan). Kidney cortex was snap-frozen in liquid nitrogen for gene and protein expression analyses, and/or embedded in 4% paraformaldehyde for histological analyses.

### Histological analysis and immunohistochemistry staining

The kidney was cut longitudinally along the long axis, fixed with 4% paraformaldehyde, embedded in paraffin, and cut into 5-μm-thick slices in a row. To determine the degree of pathological damage and fibrosis of kidney tissues, sections were stained with hematoxylin and eosin (H&E) and periodic acid-Schiff (PAS), and then the morphological changes of the kidneys were observed under a light microscope.

As previous described (Abais et al., 2014), glomerular morphology was observed and assessed semi-quantitatively. Briefly, 40 glomeruli per slide were counted and scored each as 0, 1, 2, 3 or 4. These values were respectively assigned according to the severity of sclerotic changes (0: 0%; 1: <25%; 2: 25–50%; 3: 51–75% or 4: >75% sclerosis of the glomerulus). The Glomerular Damage Index (GDI) comprised the average of these scores and was calculated according to the following formula: [(N_1_ × 1) + (N_2_ × 2) + (N_3_ × 3) + (N_4_ × 4)]/n, where N_1_, N_2_, N_3_, and N_4_ represent the numbers of glomeruli with scores of 1, 2, 3, 4, and n represents total glomeruli.

### Enzyme-linked immunosorbent assay

The levels of IL-1β and TNF-α in kidney tissues and serum were assessed by Enzyme-linked immunosorbent assay (ELISA) according to manufacturer’s instructions (MLBio, China).

### Cell culture and treatment

Mouse glomerular mesangial cells SV40-MES-13 were purchased from ATCC and cultured in Dulbecco’s Modified Eagle’s Medium (DMEM, Gibco) in low glucose (5.5 mM of glucose) supplemented with 10% fetal bovine serum (FBS, Gibco) with 1% antibiotics (100 IU/ml penicillin-streptomycin), and then grown at 37°C with an atmosphere of 5% CO_2_. Next, cells were treated with SA (10, 20, and 30 μM) dissolved in 0.1% dimethyl sulfoxide for 1 h before high glucose (30 mM, Sigma) treatment. After 24 h of culture, the supernatant and cells were collected for further analysis. To further explore the mechanism, mesangial cells were pretreated with ammonium pyrrolidine dithiocarbamate (PDTC, 50 μM, Sigma), a NF-κB inhibitor, along with SA prior to stimulating with HG for 24 h.

### Cell proliferation assay

Cell proliferation was assessed using Cell Counting Kit-8 (CCK-8) (Dojindo, Japan) and a 5-ethynyl-2-deoxyuridine (EdU) labeling/detection kit (BeyoClick™ EdU Cell Proliferation Kit with Alexa Fluor 555, Beyotime, China). For CCK-8 assay, mesangial cells were seeded in a 96-well plate exposed to high glucose (30 mM) with or without pretreatment of different concentrations of SA for 24 h. Next, 10 µl CCK-8 reagent was added to each well containing 100 µl DMEM medium and the plate was incubated for 2 h at 37°C. Finally, a microplate reader (BioTek, United States) was used to detect the absorbance at 450 nm.

For EdU assay, cells were cultured in a 12-well plate and incubated with EdU for 2 h. Trypsin was added into the microcentrifuge tube to digest the cells, which were then fixed with 4% paraformaldehyde for 15 min and permeated with 0.3% Triton X-100 for another 15 min. Next, cells were incubated with the Click Reaction Mixture for 30 min at room temperature in a dark place following the manufacturer’s instructions. Finally, cells were re-suspended in phosphate buffered saline (PBS) for flow cytometric analysis on the Beckman Coulter CytoFLEX LX (Beckman Coulter, United States). Data were analyzed using FlowJo software version 10 (Treestar).

### RNA isolation and quantitative real-time PCR

Total RNA was extracted using TRIzol reagent (Invitrogen), followed by cDNA synthesis (1,000–2,000 ng total RNA samples) using a reverse transcription kit (Yeasen, China). Quantitative real-time PCR was performed using a SYBR Green qPCR kit (Yeasen, China), and the primers were designed and synthesized by Tsingke Biotech Com (Shanghai, China). Relative mRNA expression was normalized to GAPDH mRNA and then calculated using the 2−^△△CT^ method. The primer sets used are shown in Supplementary Table S1.

### Western blot analysis

Total protein of both cultured cells and kidney cortex were extracted in RIPA lysis buffer (Beyotime) supplemented with phenylmethylsulfonyl fluoride (PMSF, Beyotime) and protein phosphatase inhibitor (Solarbio). Notably, the extraction and isolation of nuclear and cytoplasmic protein were performed according to the Nuclear and Cytoplasmic Protein Extraction Kit (Beyotime) protocol to confirm the activation of NF-kB by detecting the expression of nuclear NF-κB p65, cytosol NF-κB p65, and Lamin B. The concentration was then determined by the BCA protein assay reagent kit (Fdbio science) and the samples were homogenized in 5× SDS-sample buffer at a final concentration of 1×. Next, samples were denatured, resolved using 12% SurePAGE™ Bis–Tris Protein Gels (GenScript), and then transferred onto polyvinylidene difluoride (PVDF) membranes. After nonspecific binding was blocked with 5% nonfat dry milk in Tris-buffered saline for 1 h, membranes were incubated overnight with primary antibodies including TGF-β1 (1:1000, Abcam), Col-IV (1:1000, Proteintech), NF-κB p65 (1:1000, CST), IκBα (1:1000, CST), Lamin B (1:1000, CST), and Actin (1:2000, Abcam), at 4°C. On the next day, membranes were washed with Tris-buffered saline, incubated with horseradish peroxidase-conjugated secondary antibody (1:5000, Yeasen) at 37°C for 1 h, and visualized using Amersham Image Quant 800 imaging system (GE Healthcare). Whole cell protein expression was normalized to Actin, whereas nuclear protein expression was normalized to Lamin B.

### Assay of cellular NF-κB p65 translocation.

Cells were immunofluorescence-labeled using a Cellular NF-κB Translocation Kit (Beyotime) according to the manufacturer’s instructions. After different treatments, cells were fixed with 4% paraformaldehyde and permeabilized with 0.3% Triton X-100 for 10 min. Next, cells were washed twice with PBS containing 0.5% Tween, and incubated with a blocking buffer for 1 h at room temperature. Cells were then incubated with primary NF-κB p65 antibody overnight at 4°C, followed by a Cy3-conjugated secondary antibody for 1 h at room temperature. Finally, cells were counterstained with DAPI for 5 min and then viewed under a fluorescence microscope (400×, Leica DMi8, Germany).

### Statistical analysis

All statistical analyses were performed using GraphPad Prism v6.0 Software (United States). Data obtained are expressed as the means ± SEM. Each experiment was performed with three biological replicates. All experiments were performed at least three times. Two groups were examed *via* using two-tailed Student’s *t*-test, while multiple groups were compared using ANOVA followed by a Student-Newman-Keuls test, differences were considered to be significant at *p* < 0.05.

## Results

### SA ameliorated kidney injury in STZ-induced diabetic mice

**FIGURE 1:**
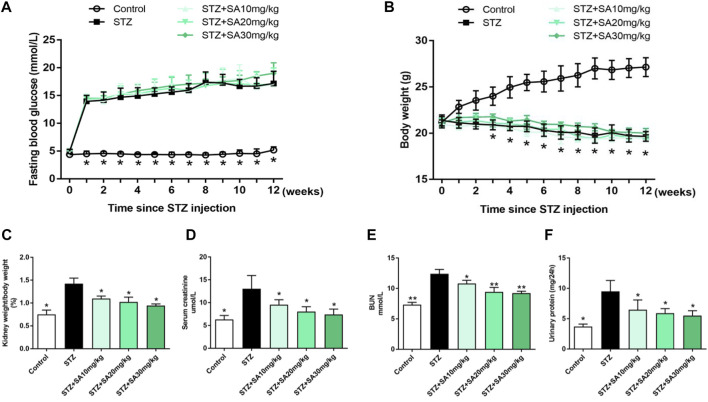
Effects of SA on metabolic and biochemical parameters in STZ-induced diabetic mice. **(A)** Fasting blood glucose levels. **(B)** Body weight. **(C)** Ratio of kidney weight to body weight. **(D,E)** The levels of serum creatinine and BUN. **(F)** 24-hour urinary protein. *N* = 5 per group. All data are shown as the mean ± SEM; *p < 0.05 and **p < 0.01 versus STZ group. SA, Sappanone A; STZ, streptozotocin; BUN, blood urea nitrogen.

As shown in Figures 1A,B, STZ treatment in uninephrectomized mice caused a considerable increase of fasting blood glucose levels and a significant decrease in body weight, however, there was no difference among the STZ-treated mice with or without SA treatment. We subsequently examined a series of indices of kidney injury to assess the beneficial effects of SA. First, mice in STZ group induced greater kidney hypertrophy indicated by an increased ratio of kidney weight/body weight, which was significantly blunted by SA administration (Figure 1C). Consistently, serum creatinine and blood urea nitrogen (BUN) were markedly elevated after STZ treatment, in contrast, the elevation of serum creatinine and BUN were significantly attenuated by SA administration (Figures 1D,E). Moreover, 24 h urinary protein was markedly increased in STZ induced mice while the level was significantly decreased after SA treatment (Figure 1F). Collectively, above results suggest that SA administration ameliorates kidney injury in STZ-induced diabetic mice.

### SA ameliorated pathological changes in the kidney and fibrosis in STZ-induced diabetic mice

**FIGURE 2:**
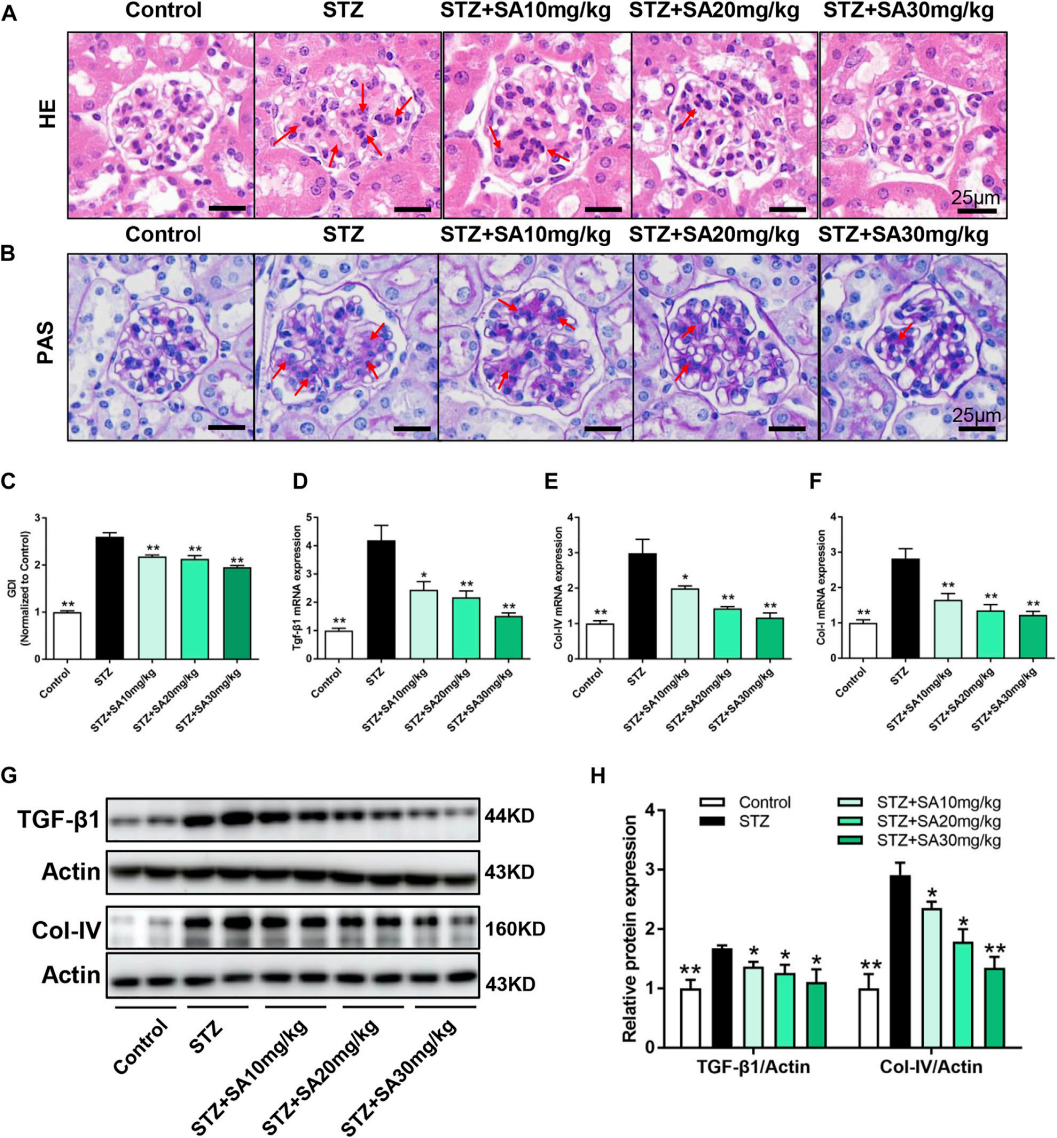
SA attenuates diabetes induced glomerular morphological changes and fibrotic process *in vivo*. **(A)** Representative photomicrographs showing glomerular structures (hematoxylin–eosin staining, 400×). Scale bar, 25 μm. **(B)** Representatives photomicrographs showing glomerular structures (PAS staining, 400×). Scale bar, 25 μm. **(C)** Summarized glomerular damage index in different groups. **(D–F)** The mRNA levels of *Tgf-β1*, *Col-IV*, and *Col-I* in kidney cortex. **(G–H)** Immunoblot and quantification for TGF-β1 and Col-IV expression. *N* = 5 per group. All data are shown as the mean ± SEM; *p < 0.05 and **p < 0.01 versus STZ group. HE, hematoxylin–eosin; PAS, Periodic Acid-Schiff; GDI, glomerular damage index; TGF-β1, transforming growth factor-β1; Col-IV, collagen-IV; Col-I, collagen-I.

H&E and PAS staining were performed to determine morphological changes in the kidney. As shown in Figure 2A, STZ-treated mice had remarkable glomerular sclerotic damage as indicated by glomerular mesangial expansion with hypercellularity, capillary collapse, and matrix deposition in glomeruli whereas these histopathological changes were markedly improved in SA-treated mice. Similar pattern of the morphological changes was observed by PAS staining (Figure 2B). The glomerular damage index is summarized in Figure 2C.

We further determine the effects of SA on kidney fibrosis under hyperglycemia. As shown in Figures 2D–F, the mRNA expression levels of the profibrotic molecules *Tgf-β1*, *Col-IV*, and *Col-I* were significantly increased in the kidney cortex of STZ-treated mice compared with control mice, in contrast, the expression of these fibrotic markers were blunted after SA administration. Similar pattern was observed on protein expression levels of TGF-β1 and Col-IV (Figures 2G,H). Taken together, the suppression of kidney fibrosis supports the possibility that SA has a beneficial effect against DKD, and the most obvious effects of SA on renoprotection is at a dosage of 30 mg/kg/d.

### SA attenuated the systemic and kidney cortical inflammatory response in STZ-induced diabetic mice

**FIGURE 3:**
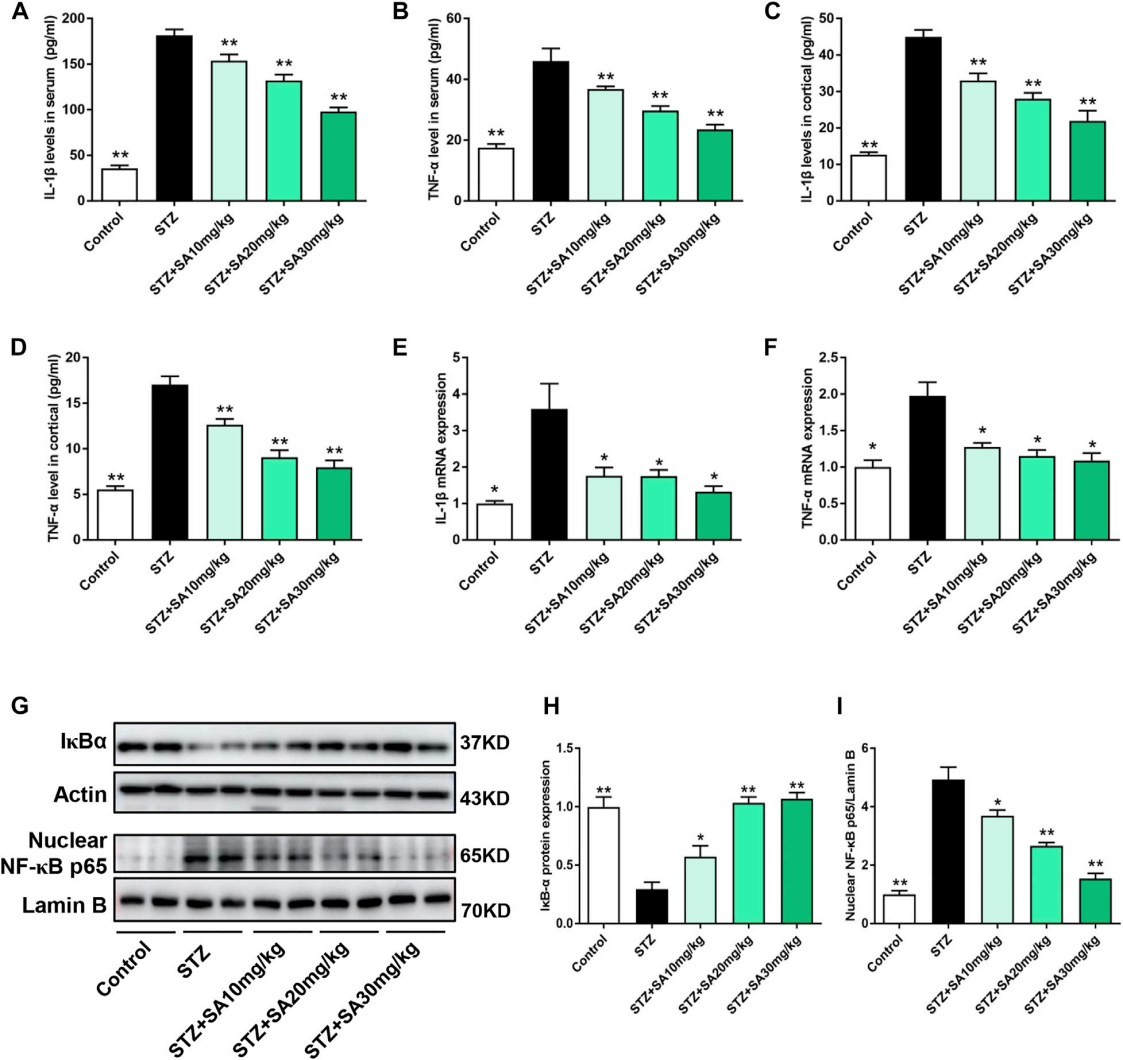
SA inhibited IL-1β and TNF-α expression as well as NF-κB activation in diabetic mice. **(A,B)** IL-1β and TNF-α excretions in serum. **(C,D)** IL-1β and TNF-α production in kidney cortex. **(E,F)** The mRNA levels of *IL-1β* and *TNF-α* in kidney cortex. **(G–I)** Western blot analysis and quantitative data for total IκBα and NF-κB p65 expression in different groups. *N* = 5 per group. All data are shown as the mean ± SEM; *p < 0.05 and **p < 0.01 versus STZ group. IL-1β, interleukin-1β; TNF-α, tumor necrosis factor-α; NF-κB, nuclear factor kappa B; IκBα, NF-κB inhibitor alpha.

It is well established that inflammation triggers the pathogenesis of DKD (Navarro-Gonzalez and Mora-Fernandez, 2008; Tang and Yiu, 2020). The present study detected increased circulating IL-1β and TNF-α levels during DKD. As shown in Figure 3A,B, different doses of SA administration alleviated IL-1β and TNF-α excretion in STZ treated mice. Consistently, SA treatment significantly inhibited the IL-1β and TNF-α production in kidney cortex (Figures 3C,D). In parallel with immunoblotting results, the mRNA levels of *IL-1β* and *TNF-α* were enhanced in kidney cortex of diabetic mice, however, SA effectively reversed above abnormalities (Figures 3E,F). As a master regulator of inflammation, the transcription factor NF-κB triggers numerous proinflammatory genes expression and plays a fundamental role in low grade sterile inflammation during hyperglycemia (Karin and Greten, 2005). Hence, we measured the protein expressions of IκBα and nuclear NF-κB p65. In STZ-treated mice, IκBα was significantly reduced while nuclear NF-κB p65 was increased in the kidney cortex compared with the normal control. In contrast, SA administration effectively reversed the pathological process (Figures 3G–I). Above results demonstrated that all three doses of SA exerted an anti-inflammatory action in STZ-treated mice.

### SA decreased HG-induced proliferation and fibrosis in glomerular mesangial cells

**FIGURE 4:**
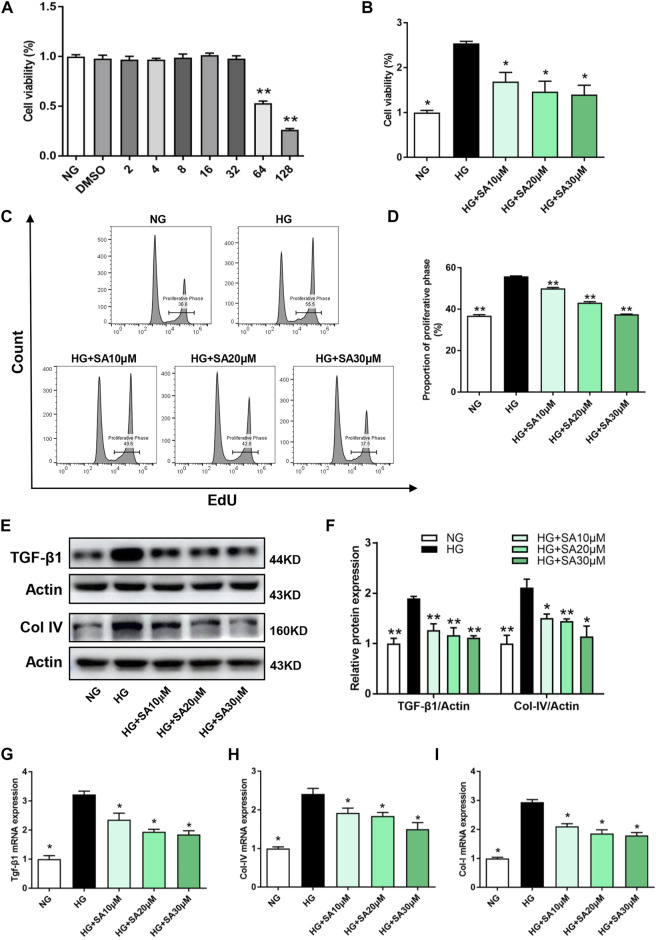
SA inhibits HG-induced proliferation and fibrosis in glomerular mesangial cells *in vitro*. **(A)** Cell viability of glomerular mesangial cells in different concentrations of SA (N = 4). **p < 0.01 vs. NG group. **(B)** Cell viability in HG group with or without different dose of SA treatment (*N* = 4). **(C)** Flow cytometric analysis of mesangial cells stained with EdU. **(D)** Percentage of proliferative rates (*N* = 3). **(E–F)** Western blot analysis and quantitative data of TGF-β1 and Col-IV expression (*N* = 4). The mRNA levels of *Tgf-β1* **(G)**, *Col-IV* **(H)**, and *Col-I* **(I)** in glomerular mesangial cells (*N* = 3). All data are shown as the mean ± SEM; *p < 0.05 and **p < 0.01 versus HG group. NG, normal glucose; HG, high glucose; EdU, 5-ethynyl-2-deoxyuridine.

In order to assess the toxicity of SA in glomerular mesangial cells, the cell viability was tested at different concentrations. Results showed that there was no significant inhibition of cell viability when glomerular mesangial cells were stimulated by SA at concentrations less than 32 μM for 24 h (Figure 4A). Thus, the dose of SA for *in vitro* experiments were optimized to 10, 20, and 30 μM. Furthermore, HG promoted proliferation of glomerular mesangial cells, which was suppressed by SA in a dose-dependent manner (Figure 4B). In addition, EdU labeling and flow cytometry were performed to determine the proportion of proliferating cells (Figures 4C,D). Western blot results demonstrated that TGF-β1 and Col-IV expression in glomerular mesangial cells increased after HG stimulation, which were significantly inhibited by SA (Figures 4E,F). Consistently, SA treatment prevented HG-induced *Tgf-β1*, *Col-IV*, and *Col-I* mRNA expressions (Figures 4G–I). Taken together, it was evident that SA could inhibit the proliferation and fibrotic processes of mesangial cells in a dose-dependent manner.

### SA inhibited HG-induced inflammatory response in glomerular mesangial cells by blocking NF-κB pathway

**FIGURE 5:**
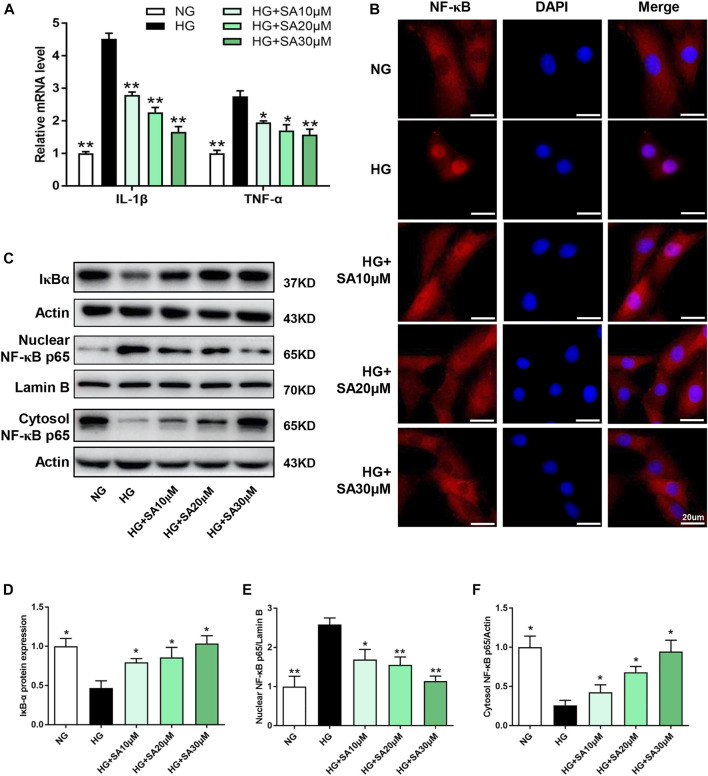
SA prevents HG-induced inflammatory responses *via* inhibiting IL-1β and TNF-α expression as well as NF-κB activation *in vitro*. **(A)** The mRNA level of proinflammatory cytokines *IL-1β* and *TNF-α* determined by reverse transcriptase polymerase chain reaction (*N* = 3). **(B)** Immunofluorescence localization of NF-κB p65 subunit (red) in mesangial cells, nuclei stained with fluorescent DAPI (blue). The images were acquired with a 40× magnification objective. Scale bar, 20 μm. **(C–F)** Immunoblot and quantification for total cell lysis IκBα, nuclear NF-κB p65 and cytoplasmic NF-κB p65 expression. (*N* = 4). All data are shown as the mean ± SEM; *p < 0.05 and **p < 0.01 versus HG group. DAPI, 4,6-diamino-2-phenyl indole.

To further investigate the effects of SA on inflammatory response *in vitro*, glomerular mesangial cells were pretreated with SA (10, 20, or 30 μM) for 1 h, followed by HG (30 mM) stimulation for an additional 24 h. *IL-1β*, and *TNF-α* mRNA expression increased significantly after HG stimulation, while SA pretreatment prevented HG-induced increase of the inflammatory cytokines in a dose-dependent manner (Figure 5A). Moreover, immunofluorescent microscopy indicated that SA prevented HG-induced nuclear translocation of p65 subunit (Figure 5B). In addition, we also assessed the effects of SA on IκBα degradation and p65 translocation in cultured glomerular mesangial cells. Our results showed that pretreatment with SA dose-dependently reversed the HG-induced degradation of IκBα, reduced HG-induced increase of nuclear NF-κB p65 levels and concomitant decrease of cytosolic NF-κB p65 levels (Figures 5C–F).

**FIGURE 6:**
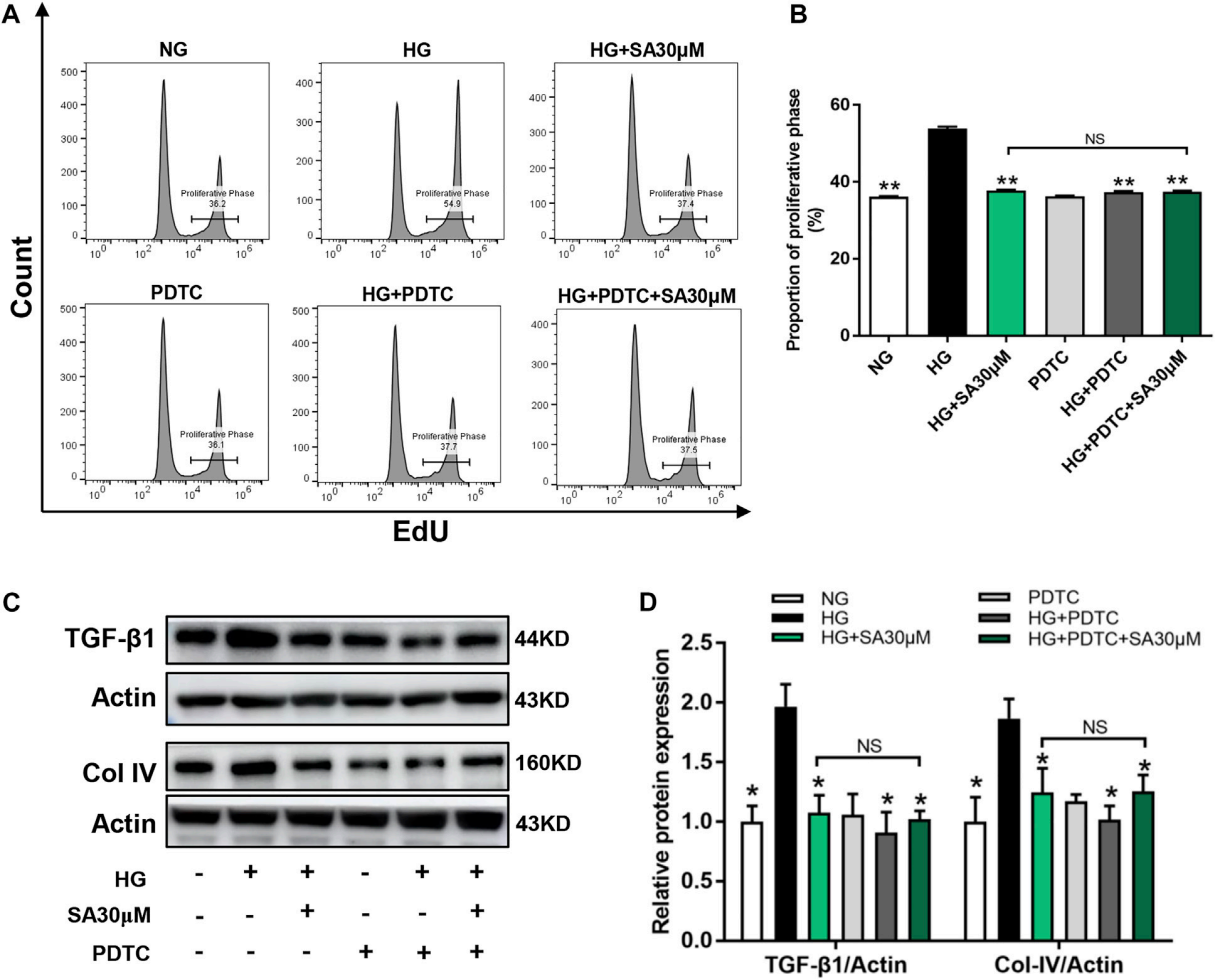
SA prevented HG-induced proliferation and fibrosis *via* inhibiting NF-κB pathway *in vitro*. Cells were pretreated with or without 50 μM PDTC, along with 30 μM SA for 1 h prior to stimulating with 30 mM HG. **(A)** Flow cytometric analysis of mesangial cells stained with EdU. **(B)** Percentage of proliferative rates (*N* = 3). **(C,D)** Western blot analysis and quantitative data of TGF-β1and Col-IV expression in treated mesangial cells (*N* = 4). All data are shown as the mean ± SEM; *p < 0.05 and **p < 0.01 versus HG group, NS: not significant. PDTC, ammonium pyrrolidine dithiocarbamate.

**FIGURE 7:**
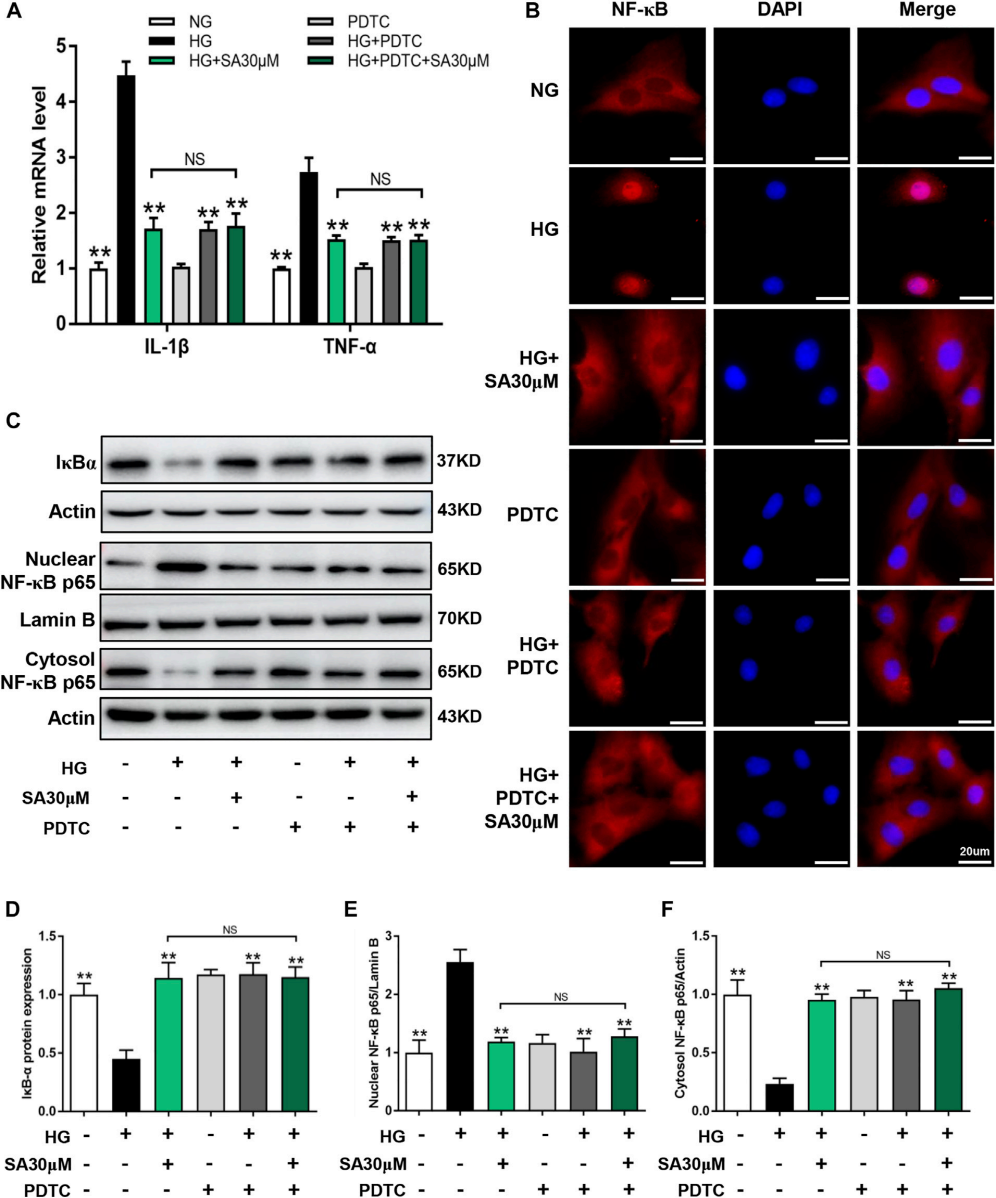
SA prevented HG-induced inflammatory process *via* inhibiting NF-κB pathway *in vitro*. Glomerular mesangial cells were pretreated with or without 50 μM PDTC, along with 30 μM SA for 1 h prior to stimulating with 30 mM HG. **(A)** The mRNA amounts of *IL-1β* and *TNF-α* in glomerular mesangial cells (*N* = 3). **(B)** Immunofluorescence localization of NF-κB p65 subunit (red) in glomerular mesangial cells, nuclei stained with fluorescent DAPI (blue). The images were acquired with a 40× magnification objective. Scale bar, 20 μm. **(C–F)** Immunoblot and quantification for nuclear NF-κB p65, cytoplasmic NF-κB p65, and total cell lysis IκBα expressions (*N* = 4). All data are shown as the mean ± SEM. *p < 0.05 and **p < 0.01 versus HG group, NS: not significant.

Next, we performed NF-κB inhibitor PDTC to further confirm that the effects of SA on HG-induced inflammatory response was mediated by the NF-κB pathway in glomerular mesangial cells. Mesangial cells were pretreated with or without 50 μΜ PDTC along with 30 μM SA for 1 h prior to HG stimulation. Although, both SA and PDTC prevented the increased proportion of cell proliferation induced by HG, there were no significant differences between the HG + SA group and the HG + SA + PDTC group (Figures 6A,B). Western blot analyses demonstrated that both SA and PDTC reduced the increased TGF-β1 and Col-IV protein levels induced by HG in mesangial cells, whereas SA combined with PDTC did not further improve the fibrosis (Figures 6C,D). Furthermore, SA and PDTC prevented the increase of *IL-1β* and *TNF-α* mRNA expression (Figure 7A), reduced the nuclear translocation of the p65 subunit (Figure 7B), reversed the degradation of IκBα, as well as the increase of nuclear NF-κB p65 levels and concomitant decrease of cytosolic NF-κB p65 levels in HG-treated glomerular mesangial cells (Figures 7C–F). However, compared with a single PDTC treatment, the combination of SA and PDTC did not further prevent the inflammatory injury caused by high glucose. Above results suggested that SA blocked HG-induced inflammatory process by suppressing the NF-κB pathway.

## Discussion

Current study evaluated the reno-protective effects and explored underlying molecular mechanisms of SA in STZ-induced diabetic mice. To date, the study provided strong evidence that SA prevented kidney injury during hyperglycemia. It is noteworthy that hyperglycemia is the foremost etiological factor of DKD, which induces profibrotic responses in kidneys of diabetic patients, eventually promoting glomerular sclerosis and interstitial fibrosis (Wu et al., 2020; Zhao et al., 2020). In our present study, SA treatment dose-dependently reduced kidney injury in STZ-treated mice, as evidenced by a decrease in ratio of kidney weight to body weight, serum creatinine, blood urine nitrogen and 24-h urinary protein. Consistently, SA administration ameliorated hyperglycemia induced glomerular morphological changes in STZ-treated mice. In addition, we also explored the beneficial effects of SA on HG-treated glomerular mesangial cells *in vitro*. Under diabetic conditions, glomerular mesangial cells are activated, leading to cell proliferation and excess glomerular ECM deposition in the mesangial region. Besides, glomerular mesangial cells also secrete various inflammatory cytokines, adhesion molecules, chemokines and enzymes in response to HG stimulation (Wei et al., 2021). Previous studies have elucidated that ECM accumulation and glomerular mesangial cells proliferation are known to be intimately associated with diabetes induced glomerular hypertrophy and sclerosis (Tung et al., 2018). In the present study, we found that SA treatment weakened HG-induced TGF-β1 and Col IV expression in glomerular mesangial cells. Consistently, the flow cytometry results revealed that SA treatment reversed glomerular mesangial cells proliferation during hyperglycemia. Above results demonstrated that SA administration alleviated diabetes induced kidney fibrotic process *in vivo* and *in vitro*.

Several inflammatory cytokines participate in the pathophysiology of DKD. Among which, IL-1β and TNF-α are considered to be the representative regulators of inflammation. It was reported that IL-1β in the kidney increased in the rodent animal models of DKD, which was associated with increased production of adhesive molecules, such as intercellular adhesion molecule 1 (ICAM-1) and vascular cell adhesion protein 1 (VCAM-1) (Sassy-Prigent et al., 2000; Navarro-Gonzalez et al., 2011). Similar to IL-1β, TNF-α also exerts various biological effects in DKD, including its direct cytotoxicity to kidney resident cells as well as activation of cell pathways leading to apoptosis and necrosis (Navarro-Gonzalez et al., 2011). Furthermore, TNF-α promotes oxidative process in mesangial cells *via* activating NADPH oxidase, leading to overproduction of reactive oxygen species (Koike et al., 2007). Recently, SA was reported to inhibit the production of inflammatory cytokines: TNF-α and IL-1β in the kidney of cisplatin-induced injury (Branstetter et al., 1989), which was in coincidence with our results in DKD.

Although several innate immune pathways have been postulated in the pathogenesis and progression of DKD, NF-κB signaling pathway is the most well-defined in a sterile glomerular and interstitial inflammatory process (Guijarro and Egido, 2001; Yang et al., 2019; Tang and Yiu, 2020). For instance, it was reported that NF-kB inhibitor reduced macrophage infiltration in the kidney, decreased production of inflammatory cytokines, and consequently reversed kidney dysfunction in diabetic rats (Yang et al., 2019). In addition, PDTC, one of NF-κB inhibitors, prevented IκB phosphorylation, thereby blocking NF-κB translocation to the nucleus, which ultimately reduced the expression of downstream cytokines (Liao et al., 2017). Thus, we continued to explore whether SA exerted beneficial actions *via* inhibition of NF-κB signaling pathway. As expected, SA administration significantly reversed IκB degradation and p65 nuclear translocation in STZ treated mice and HG treated glomerular mesangial cells. Furthermore, although blockage of NF-κB by PDTC or SA significantly attenuated abnormal proliferation and fibrotic molecules expression in HG treated mesangial cells, the combination of SA and PDTC did not further improve the phenomena. Above results demonstrated that SA indeed prevents kidney injury *via* inhibition of NF-κB signaling pathway.

Studies in some rodent models of DKD have shown that immunosuppressive drugs exhibited reno-protective effects. For instance, mycophenolate mofetil (MMF), a classic inosine monophosphate dehydrogenase (IMPDH) inhibitor, was reported to suppress proteinuria and attenuate glomerular sclerosis and interstitial fibrosis in STZ-treated rats. Similar results were reported in an experimental model of T2DM, in which MMF treatment resulted in reduced glomerular and tubulointerstitial inflammatory cell infiltration as well as decreased proteinuria. Despite these promising results in rodent animal models, immunosuppressive interventions are still not a clinical therapeutic option in real patients with DKD because of potential adverse-effects. Interestingly, Tu et al. (Liao et al., 2017) found that SA exerted anti-neuroinflammatory effects because of its highly selective inhibition of IMPDH2. However, other previous studies have demonstrated that SA directly inhibited NF-κB activation (Lee et al., 2015; Kang et al., 2016). Diverse mechanisms of SA may be dependent on different experimental models. In our present study, no significant immunosuppressive effects such as infectious disease or diarrhea were observed in the whole period of SA-treated mice. Taken together, we speculate that SA may have a direct modulation of NF-κB pathway in STZ-induced diabetic mice, which is worth further exploration in another study.

## Conclusion

In summary, the present study demonstrated that SA prevents DKD by ameliorating glomerular pathological changes, decreasing biological parameters, attenuating inflammatory molecules production and secretion, as well as alleviating fibrotic process. *In vitro*, we also found that SA ameliorates inflammatory response and fibrosis in HG-treated glomerular mesangial cells *via* inhibiting NF-κB signaling pathway. These results are promising and may provide new evidence for the potential application of SA in the treatment of DKD.

## Data Availability

The raw data supporting the conclusion of this article will be made available by the authors, without undue reservation.

## Abbreviations

BUN, blood urea nitrogen; CCK-8, cell counting kit-8; Col-IV, collagen IV; Col-I, collagen I; DAPI, 4,6-diamino-2-phenyl indole; DKD, diabetic kidney disease; DMEM, Dulbecco’s Modified Eagle’s Medium; ECM, extracellular matrix; EdU, 5-ethynyl-2-deoxyuridine; ELISA, enzyme-linked immunosorbent assay; FBS, fetal bovine serum; GAPDH, glyceraldehyde-3-phosphate dehydrogenase; GBM, glomerular basement membrane; GDI, glomerular damage index; H&E, hematoxylin and eosin; HG, high glucose; IL-1β, interleukin-1β; IκBα, NF-κB inhibitor alpha; NF-κB, nuclear factor kappa B; NG, normal glucose; PAS, periodic acid-Schiff; PDTC, ammonium pyrrolidine dithiocarbamate; qPCR, quantitative real-time PCR; SA, Sappanone A; STZ, streptozotocin; TGF-β1, transforming growth factor-β1; TNF-α, tumor necrosis factor-α.
